# Assessment of the effect of climate changes in the Late Pleistocene and Holocene on niche conservatism of an arvicolid specialist

**DOI:** 10.1038/s41598-018-28000-0

**Published:** 2018-06-28

**Authors:** Elena Castellanos-Frías, Nuria García, Emilio Virgós

**Affiliations:** 1 Dpto. Geodinámica, Estratigrafía y Paleontología, Facultad de Ciencias Geológicas, Universidad Complutense de Madrid, ES-, 28040 Madrid, Spain; 2 Dpto. Biología y Geología, Física y Química Inorgánica, ESCET, Universidad Rey Juan Carlos, C/ Tulipán s/n, ES-, 28933 Móstoles, Madrid Spain

## Abstract

Climate change is not only evident, but its implications on biodiversity are already patent. The scientific community has delved into the limitations and capabilities of species to face changes in climatic conditions through experimental studies and, primarily, Species Distribution Models (SDMs). Nevertheless, the widespread use of SDMs comes with some intrinsic assumptions, such as niche conservatism, which are not always true. Alternatively, the fossil record can provide additional data to solve the uncertainties of species’ responses to climate change based on their history. Using a combined environmental (niche overlap indices) and geographical approach (temporal transferability of SDMs), we assessed the niche conservatism of *Microtus cabrerae* throughout its evolutionary history: the Late Pleistocene and the Holocene. The set of analyses performed within this timeframe provides a broad view pointing to a shift in the realized climatic niche of the species. Specifically, *M. cabrerae* exhibited a broader niche during glacial times than interglacial times, expanding towards novel conditions. Hence, the species might have developed an adaptive ability, as a consequence of mechanisms of local adaptation or natural pressures, or just be preadapted to cope with the novel environment, due to expansion into an unfilled portion of the niche. Nevertheless, the more restricted realized niche during last interglacial times reveals that the species could be close to its physiological limits.

## Introduction

Global climate change is evident and its patent effects on biodiversity include changes in range boundaries, species abundances and phenological shifts^1,2^. Nevertheless, other effects might be important, since genetic diversity might also be affected both at the organism and biome level (animal communities)^3,4^. Consequently, a wide array of experimental studies has tried to elucidate the limitations and capabilities of the species to identify their physiological thresholds (*e.g*. Hovenden, *et al*.^5^, Neuwald and Valenzuela^6^).

Species Distribution Models (SDMs) have been used to associate species occurrence with environmental predictors through mathematical algorithms to estimate the species’ climatic niche^7^. Although SDMs are widely used, some basic assumptions such as the ecological niche conservatism (“niche stability” *sensu* Nogues-Bravo^8^) have been scarcely tested. Niche conservatism refers to the preservation of the ecological traits linked to the species’ niche^9^. The conservation of the ecological niche of species in space and time remains controversial^10–13^. The mechanisms of local adaptation to marginal conditions or natural pressures could induce evolutionary processes in the long-term^14,15^, or just expansion into a previously unfilled portion of the niche^16^, therefore, some degree of variation in the species’ niche might be expected^17^. However, whenever SDMs are aimed to forecast future climate change impacts, the niche conservation assumption implies a static snapshot of the species’ environmental niche that ultimately underestimates the real abilities of organisms to cope with new environmental conditions. Specifically, SDMs do not consider the species’ phenotypic plasticity or preadaptation to non-analogue climates, so their accuracy is reduced^16^. Moreover, a good performance of the models requires the stability of the biotic interactions and the dispersion ability shaping the realized niches of the species^16,18^. Some of these drawbacks can be palliated using other methodological approaches, such as community-level models (CLMs)^19^ or multi-temporal models to approximate the fundamental niche^18^. Alternatively, the use of fossil records can assist to study the responses of the species to climate changes. Fossils result a useful and rich source of information about the grade of evolution within lineages or species and thus, conservation or shifts in niches. In fact, the SDM approach and paleobiological data can be combined as a tool to assess current distribution patterns and define conservation guidelines^20^. The most recent part of the Quaternary (Late Pleistocene and Holocene) was characterized by periods of cold and warm climate conditions, such as the Last Interglacial Period (LIP), the Last Glacial Maximum and the Mid-Holocene. The projection of current distributions models to those analogous, warmer or glacial climates can reveal suitable territories for the species under these scenarios^21^. The subsequent evaluation of these models with an independent data set of fossil record may clarify the survival strategies and biological thresholds of the species (*e.g*. McGuire and Davis^22^, Worth, *et al*.^23^).

Interpretation problems can arise when SDMs are projected under other scenarios, since the geographical space is not likely to keep the same environmental range of values along the time periods considered. Hence, it is important to discern the niche-biotope duality to discern between the environmental and the geographical space occupied by the species^24^. The multidimensional environmental space can be summarized using techniques such as multivariate statistics to identify changes in niche features in space or time (*e.g*. Broennimann, *et al*.^25^). Therefore, a combined niche-biotope approach can provide complementary data to assess niche dynamics^24^.

The sensitivity of small mammals to local environmental changes makes them good indicators of climate conditions^26^. Furthermore, their short life cycles make them suitable for the study of responses to either subtle or pronounced environmental changes. Small mammals reflect accurately any variation in niche conditions^27^. In this study, we assess a possible shift in the climatic niche of the Cabrera vole (*Microtus cabrerae*), an endemic arvicoline of the Iberian Peninsula, accounting for its evolutionary history. *M. cabrerae* has a reduced distribution range compared to its fossil record^28^, which included locations in south-eastern France and north-eastern Spain. However, the species distribution is currently limited to a fragmented area in central-south Portugal and Spain^29^. Agriculture combined with urban infrastructures have been identified as the main causes of the current population decline^17,30,31^. Nevertheless, the effect of climate on the niche of the Cabrera vole’ seems to play an important role^32^. The species only inhabits in perennial grasses of *Agrostis castellana* and/or *Stipa gigantea* and rush beds in open areas with a high water table^30,31^. Therefore, the species could be expected to expand its favourable habitats under humid conditions, while xeric conditions could decrease habitat suitability. Araujo, *et al*.^33^ and Mestre, *et al*.^34^ predict the species to experience a northward range shift and, a strong contraction of the southern portion of its distribution. As this habitat-specialist species has persisted despite adverse climate changes, *M. cabrerae* may have some adaptive ability to face the restrictions. We hypothesized that cold conditions (*e.g*. during the Last Glacial) might not be as limiting to the species as a warm, arid scenario (*e.g*. during the Last Interglacial). Therefore, we aim to elucidate on the Cabrera vole’s ability to cope with climate change since the beginning of its history and to clarify whether climate alone explains the species’ current distribution. Using an environmental and geographical approach we assessed the niche dynamics of *M. cabrerae* that have occurred during the climate changes since the Late Pleistocene. Specifically, we projected a current SDM to three paleoclimate scenarios (and vice versa) and measured the climatic overlap of current and paleo-niches to evaluate the niche conservatism of the species.

## Results

### Climatic niche using a geographical approach

Among the modelling algorithms, the Random Forest (RF) model had the best performance in predicting current species occurrences (Table 1).This algorithm explained the 53% of the variance despite only considering climatic variables. Although all models had a good current predictive ability, none accurately located the fossil records of Cabrera voles when projected into paleoclimate scenarios. Model validation with the fossil record (independent data) provided poor area-under-the-curve (AUC) values (range: 0.302–0.670), indicating that the models did not perform better than a random prediction.

**Table 1:** Modelling algorithms used to identify the paleo-niches of *M. cabrerae* from the current distribution. Models are calibrated under current conditions and projected to current and paleoclimate scenarios. Model performance is evaluated through a double approach: the statistical assessment of the model with the variance explained and the predictive ability of each model in current (internal validation) and past scenarios (external validation) with AUC.

Model	Dataset	Explained variance		AUC			
		Variance	Gain	Current	MIS1	MIS2	MIS5e
GLM	pseudo	0.373		0.878	0.662	0.613	0.402
RF		0.530		0.967	0.670	0.414	0.302
Maxent	bg		0.594	0.836	0.634	0.598	0.462
GLM		0.325		0.849	0.653	0.625	0.408

pseudo: pseudoabsences; bg: background.

Both GLM deviance (R function D-squared, library modEva^83^) and RF variance (R library randomForest^73^) are expressed as a decimal. Whereas, gain value from Maxent defines the exponent to generate the average likelihood of presences, therefore the maximization of gain results in a model that best discriminates presence and background data^77,78^. The AUC of the current scenario was obtained from a bootstrapping evaluation; on the other hand, the AUCs from past transferabilities were calculated using fossil records as independent occurrence points.

**Figure 1:**
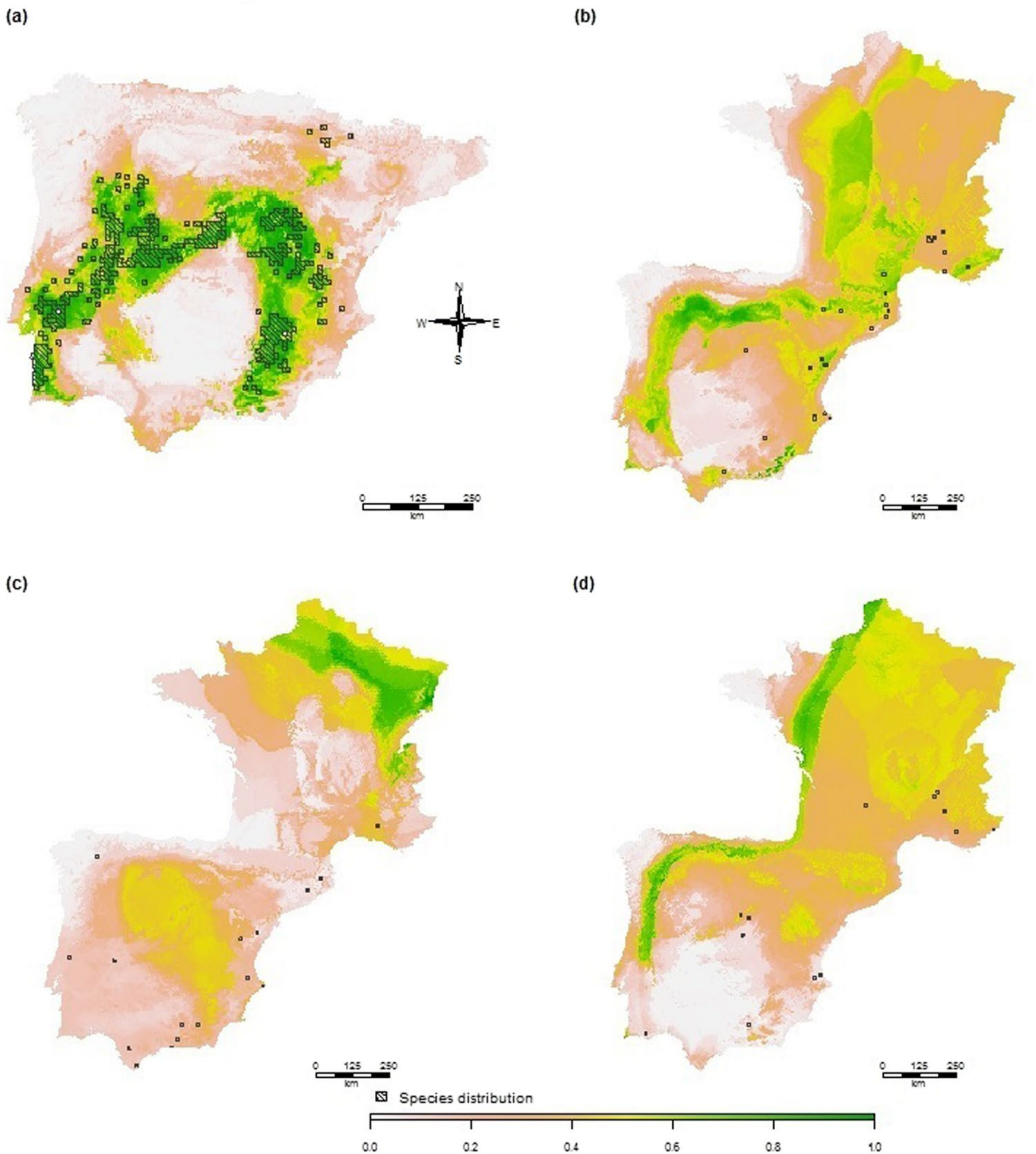
Random forest model projection to (**a**) current, (**b**) Mid-Holocene, (**c**) Last Glacial Maximum and (**d**) Last Interglacial Period climatic scenario. The shaded areas represent current occurrences (**a**) or fossil record of *M. cabrerae* for each epoch (**b**–**d**). Model is calibrated with the current distribution, thus it is restricted to the Iberian Peninsula, however the paleo-distribution included also France territories. Figure was created in R v3.3.2^72^ (https://www.R-project.org/).

**Figure 2:**
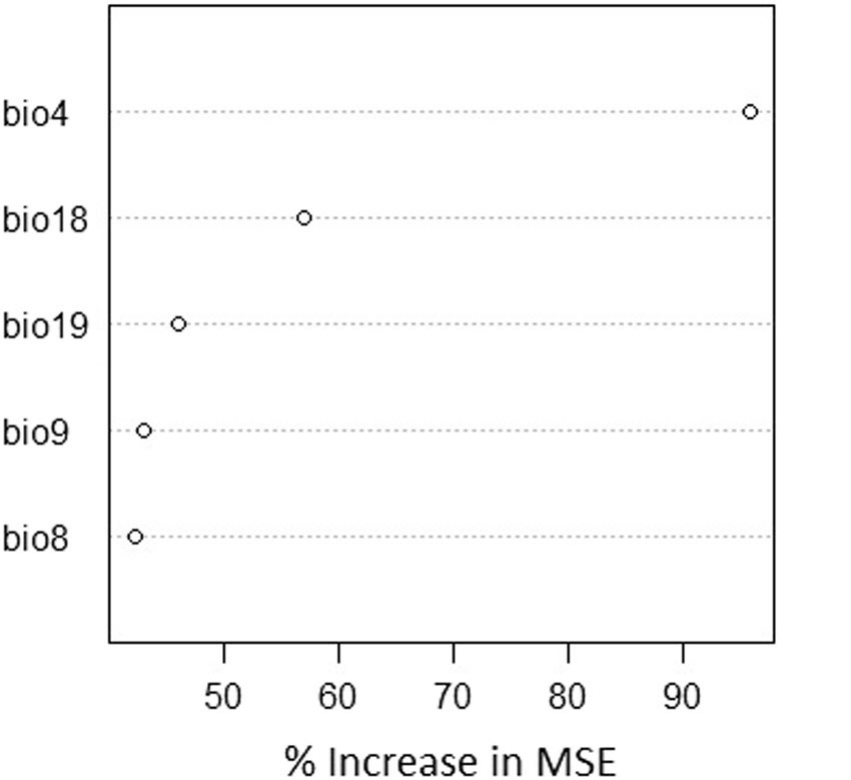
Importance of bioclimatic variables used in the random forest SDM from the geographical approach (R function varImpPlot, library randomForest^73^). The percentage of increase in mean square errors (MSE) of model predictions is collected for each variable when that variable is randomly permuted, hence its importance in the accuracy of the model. Bioclimatic variables: temperature seasonality (bio 4), mean temperature of the wettest quarter (bio 8), mean temperature of the driest quarter (bio 9), precipitation in the warmest quarter (bio 18) and precipitation in the coldest quarter (bio 19).

All models were accurate in predicting the location of suitable territory currently used by the species. Only geographical projections of the best algorithm (RF) are summarized in Fig. 1, although the remaining models followed a similar trend. Temperature seasonality was the most explanatory variable in the models, since its random permutation had the greatest effect in the model predictions (Fig. 2); followed by precipitation variables. The species showed a preference for areas with a marked Mediterranean climate, as Cabrera voles occurrences were concentrated in areas with high temperature seasonality values (Supplementary Fig. S1) up to a threshold (temperature seasonality values above *ca*. 6700 units) at which occurrence was again minimized. Consequently, the current niche predicted for the species was the central, south-western and south-eastern strips of the Iberian Peninsula (Fig. 1a). The most northern populations (Fig. 1a) were not accurately identified with any of the current SDMs, since the values of the bioclimatic variables in this area were outside the range commonly preferred by the species (Supplementary Fig. S1). Some degree of spatial transferability of Cabrera voles beyond their current distribution limits was found in south-eastern France, as shown in Supplementary Fig. S2.

In contrast, the transferability of the models to past conditions revealed that the most appropriate areas for the Cabrera voles were the north-eastern strip under Atlantic influence, Northern France and some territories under the effect of the Mediterranean Sea, depending on the epoch (Fig. 1b–d). These areas of high climatic suitability had the same range of temperature seasonality as that preferred by the species in its current niche. However, when temperature seasonality exceeded the calibration thresholds of the current model (Supplementary Fig. S1), species suitability in the paleo-scenarios was extrapolated to minimum values (according to the current biological response of the species). The most dissimilar variable analysis (MoD) showed the areas with non-analogous conditions in the Marine Isotope Stage 1 or MIS1, and especially in MIS5e (Supplementary Fig. S3). Therefore predictions in these areas would be speculative. Conversely, MoD revealed analogous environmental conditions between MIS2 and current climate scenarios. Beyond these non-analogous areas, hindcastings made some inaccurate predictions, such as the identification of northern France as the niche of this Mediterranean species during the coldest period (the Last Glacial Maximum: LGM).

The cross-projections of algorithms (Table 2) followed a similar trend to the models calibrated under current conditions and projected to the past. In general, the models calibrated with the pseudoabsence dataset had higher AUC values in the internal evaluation (range: 0.813–0.958 versus 0.749–0.860 of the background dataset). However, independent validations with current occurrences reflected poor transferability in projections from the past to the present, as they were no different from random projections.

**Table 2:** Cross-projections. Modelling algorithms were calibrated in each paleoclimatic scenario and evaluated with AUC from the bootstrapping process (AUC of calibrated models). The evaluation of transferred models from paleo-scenarios to current climate was measured with AUC values using current occurrences as independent points (AUC of projection to current climate).

Model	Dataset	AUC (Calibrated Models)			AUC (Projection to Current Climate)		
		**MIS1**	**MIS2**	**MIS5e**	**MIS1**	**MIS2**	**MIS5e**
GLM	pseudo	0.925	0.813	0.816	0.599	0.553	0.589
RF		0.955	0.899	0.958	0.560	0.561	0.593
Maxent	bg	0.801	0.799	0.749	0.491	0.601	0.597
GLM		0.860	0.799	0.766	0.578	0.566	0.587

pseudo: pseudoabsences; bg: background.

### Climatic niche using an environmental approach

**Figure 3:**
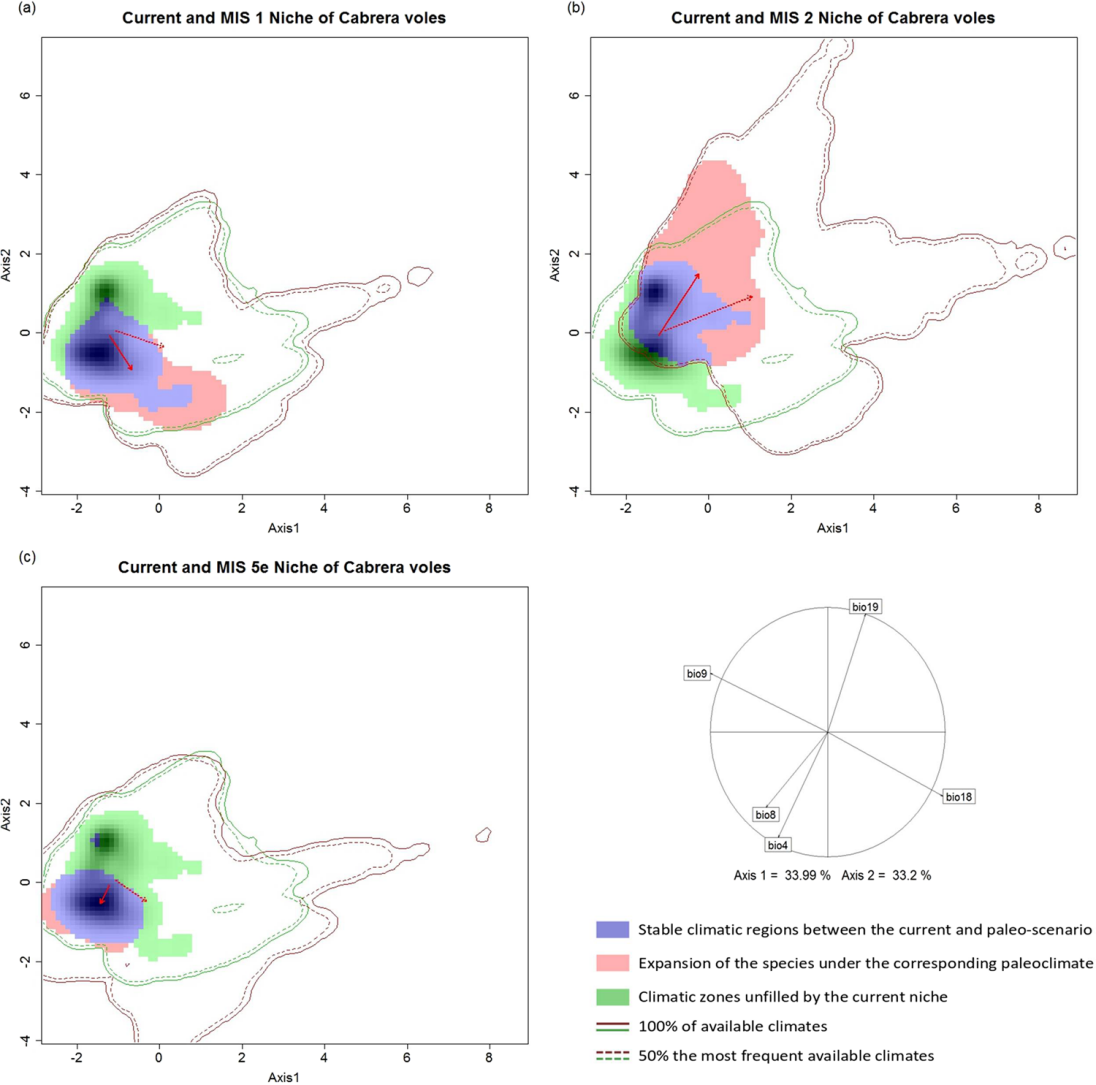
Climatic occupancy of *M. cabrerae* under Current and Paleo-climate: (**a**) MIS1; (**b**) MIS2 and (**c**) MIS5e periods. The arrows visualize the shift of the niche centroids between the current and paleo-scenario: continuous lines represent the shift in the centroid of current and paleo-distribution, while the dashed line is related to current and paleo extent. The correlation circle collects the contribution of bioclimatic variables to PCA axes.

The global climatic space of the four scenarios (current, MIS1, MIS2 and MIS5e) was compiled through two PCA (principal components analysis) axes (Fig. 3). The variables of precipitation and temperature of warmest and driest quarter explained 33.9% of the variability, while the precipitation of coldest quarter, the temperature seasonality and the mean temperature of wettest quarter summarized 33.2% of the variability. The climatic space of MIS1 and the current scenario was collected in Fig. 3a. The niche of the species under the MIS1 climate occupied areas with higher precipitation in the warmest quarter and lower precipitation in the coldest quarter. Similar results were obtained in MIS5e (Fig. 3c). Niche stability was especially high in MIS5e with a value of 98% (Table 3); while the expansion ability of Cabrera voles under the warmer scenario was reduced to minimum values (2%, Table 3). On the other hand, MIS2 showed low stability in the environmental niche (55%, Table 3) and a niche expansion (45%, Table 3), indicating that *M. cabrerae* could have expanded its niche to a novel climatic framework. In MIS2 (Fig. 3b), the realized niche had higher mean temperature and precipitation during the driest and the coldest quarter, respectively, lower values of temperature seasonality and lower precipitation in the warmest quarter.

**Table 3:** Niche overlap comparisons between current and paleo- scenarios of *M. cabrerae*. The statistical significance of niche overlap to evaluate the hypothesis of niche equivalency and the hypothesis of niche similarity were conducted with randomization tests. For niche equivalency, a significant result implies that the overlap between the current and the paleo-scenario is less than would be expected if niches were identical. The significance for similarity tests means that current and paleo-niches are more similar than would be expected from a random distribution of the species created inside the paleoclimate-scenario (CC-Paleo) or created inside the current climate (Paleo-CC).

Scenario	Niche Overlap	Overlap index			Equivalency test	Similarity test	
	*D*	Expansion	Stability	Unfilling		CC to Paleo-	Paleo- to CC
CC - MIS1	0.166	0.257	0.743	0.316	**	*ns*	*ns*
CC - MIS2	0.151	0.451	0.549	0.169	**	*ns*	**
CC - MIS5e	0.480	0.019	0.981	0.312	**	**	**

CC: Current Climate.

*ns* (non-significant): p > 0.05; *p ≤ 0.05; **p ≤ 0.01.

Concerning the overlap of the environmental niche of paleo-scenarios and the current niche, none of the assessed paleo-niches were climatically equivalent to the current realized niche, as it is shown by the low Schoener’s D values obtained (p < 0.05; Table 3). The results of the similarity test were not homogeneous throughout the three paleo-scenarios (Table 3). Since the similarity hypothesis could not be rejected under the MIS1 scenario, niche overlap between the two temporal settings could be explained by regional similarities. Under the Current Climate-MIS2 scenarios, this hypothesis could only be partially rejected, as the paleo-niche of the species under cold MIS2 was not as similar to the current one as expected based on the environmental similarity between the two periods. However, this pattern was not observed from current climate to MIS2 scenario. Despite the non-equivalency between current and MIS5e niches, both were more similar to each other than would be expected by chance.

## Discussion

The geographical and environmental assessments of the niche of the Cabrera vole illustrated a shift in its realized niche throughout its history. From an environmental perspective, the analysis revealed a lack of equivalency between current and paleo- niches, and therefore, reciprocal niche predictions from either range were unsuccessful. On the other hand, the similarity analyses clarified the response of *M. cabrerae* to the succession of climate changes from the Late Pleistocene until the present, indicating the expansion of the species’ niche to novel climatic conditions. Thus, subtle variations in the MIS1 climate were enough to drive changes in both the niche centroid and the niche envelope. Consequently, the LGM led to a major niche shift and the species was pushed towards areas of marked Mediterranean conditions. Laplana and Sevilla^28^ identified a contraction in the geographical distribution of the species with a movement towards lower latitudes, which supports our results of a shift to wetter and warmer territories. Conversely, even though the niche during MIS5e was not identical, the hot climatic conditions make the realized niche in that period more similar to the current niche than the surrounding locations. MIS5e was characterised by an average increase in temperature of 3.8 °C and an average decrease in precipitation of 16% with current climate during the driest and warmest quarters. These arid conditions promoted a restricted realized niche compared to the current one. Assessments of the current status and threats to Cabrera vole populations have found that Mediterranean summers, with their high temperatures and low or no rainfalls, impose high restrictive conditions which act as a bottleneck, reducing population densities of this small mammal^30,32,35,36^. Such climatic conditions of the current and Last Interglacial Period could be close to the species’ physiological limits^32,37^. These conditions could have relegated the species’ niche to specific habitats which could ensure the necessary environmental requirements that could not be satisfied by climate (*e.g*. areas of high phreatic level or areas in mountain foothills)^28,38^.

From the geographical approach, SDM transferabilities were not able to locate the realized niche of *M. cabrerae* for any of the scenarios based on the fossil record. The main drawback in these temporal transferabilities is the non-analogue conditions between the current and paleoclimatic scenarios for some variables like temperature seasonality. Even though temperature seasonality values fall outside the calibration ranges, it is a very important variable as shown by both by the SDMs and the biology of the species^36^, and, therefore, its use is justified. Excluding these speculative predictions, the suitable areas predicted by hindcasting are very distant from the fossil record. Furthermore, the inaccurate current predictions from the SDMs cross-projections support that the relationship of the species with the environmental variables could have changed. This shift could have affected the set of bioclimatic variables defining the realized niche of Cabrera voles, as detected in some biological invasions of plants^12^ and temporal transferabilities of mammals^39^. The use of additional biotic variables such as habitat type, degree of fragmentation of the territory or distribution of other species could have improved SDM transferability^40^. However, these variables would have to be estimated from extrapolated data for the paleo-scenario, which would make the transferability of SDM projections less accurate. Despite using a well-calibrated and complete fossil record database, the use of fossil records can result in potential biases, as the records may not be representative of the species’ distribution. For instance, most Cabrera vole fossils in karsts of calcareous formations are from mountains of alpine origin, and there has traditionally been less interest in the study of small mammals^28^. In addition, the number of records from the Last Pleistocene sites is quantitatively lower than in those with recent chronology^28^. This could lead to inaccurate predictions of the species paleo-niches, and these limitations could be skewing our results.

Fløjgaard, *et al*.^21^ and Vega, *et al*.^41^ used SDM transferability to properly identify the niches of a set of North-Central European small mammals during the LGM, assuming niche conservatism. Conversely, McGuire and Davis^22^ inferred a niche shift in three out of five evaluated *Microtus* species since the last notorious climate change, the LGM in North America. Likewise, Guralnick and Pearman^42^ identified a change in the niche of two small mammals species from flatland territories, while a set of mountainous species did not show a niche shift. However, the lack of transferability of the SDMs of five mammal species to the North American LGM led Davis, *et al*.^43^ to reject the flatland-mountain hypothesis. Accordingly, studies on microtines have revealed varied responses and grades of niche conservatism dependent on taxa, environmental context or the temporal-frame^44^.

The poor performance of SDM transferability has generally been interpreted to support niche shift^12,22,23,42,43^. The grounds for these niche shifts may be mainly attributed to some adaptation ability, a truncation of species’ fundamental niche or the absence of equilibrium between the species’ distribution and climate^16,18,45,46^. Microtines are a highly diverse group of mammals with high karyotypic variability and a rapid evolution rate, contrary to what could be expected due to their phenotypic homogeneity^47–49^. According to Triant and DeWoody^49^, new *Microtus* species may evolve every 30000 years; consequently, changes in their fundamental niches could be expected. However, the identification of such adaptive processes requires mechanistic approaches with experimental measurements^45^. Our methodological framework is unsuitable to rule out phenotypic plasticity or preadaptation, as a consequence of the truncation of the fundamental niche of the species. The niche truncation involves a hiding of the species’ range to climatic tolerance^50^; thus, it may explain the expansion to novel climatic conditions of the current and paleo- realized niches. Despite considering several factors, Veloz, *et al*.^16^ pointed out truncation as the most probable cause to explain the failure in current niches predictions of fossil-pollen data from 21 to 15 kyr. B.P. In fact, these authors conclude that the realized niche only covers a temporal subset of the suitable environmental conditions of the taxon. Likewise, shifts in niches may be caused by changes in positive or negative biotic interactions or in dispersal limitations (*i.e*. lack of species-climate equilibrium^8^) of *M. cabrerae* since the Late Pleistocene. Pearman, *et al*.^46^ revealed the importance of biotic interactions as modeller of the realized paleo-niche of some plant species (larger influence than climate). Some authors consider that this lack of success could also be attributed to other factors such as excessive model complexity or overfitted models^19,44^, inaccuracies in paleoclimate reconstructions^22^, vagueness in fossil dating^43^ or not considering other factors than temperature and precipitation^40,43^. Part of the SDM’s limitations could be overcome using a wider approach based on CLMs^19^ or multi-temporal approaches to estimate fundamental niches^18^. CLMs solve the model overfitting because they include multi-taxa data, which provides a better prediction in non-analogue climates or communities^19^.

Under the current timeframe, all SDMs had good performance and properly located the occurrences of the species. According to Garrido-García, *et al*.^51^, the distribution of *M. cabrerae* is arranged in four unconnected population cores. However, our models found high climatic connectivity between occurrences, which may be due to some biotic interaction or anthropogenic disturbance. Spatial transferability beyond current distribution limits reveals areas of climatic suitability in south-eastern France, even though the species is not currently distributed there. The fossil record shows that Cabrera voles inhabited this territory from the Late Pleistocene until quite recent times, as the last records in France dated from *ca*. 1900–2000 yr. B.P.^52^. Previous studies have reported a historic contraction in the distribution of the species and its disappearance from France as a result of climate, interspecific interactions and/or an intensification of anthropogenic impacts^28,38,53^. Nevertheless, the results suggest that climate is not the main cause of extinction in the French territory. Furthermore, the Pyrenees mountain chain was not an impassable barrier for the species as it was not for similar microtines of northern latitudes like *Microtus oeconomus*. The fossil record shows that this species crossed the Pyrenees during cold MIS2 from northeastern Europe and reached the Central mountain range of the Iberian Peninsula^28^, searching for a suitable climate. So, even though the less favourable climate could explain the contraction in Cabrera vole distribution, we consider the species might be able to recover a territory with current climatic suitability, as it did during the events of past climate change. On the other hand, southeastern France is currently inhabited by *Microtus agrestis*, *Microtus arvalis* and *Arvicola sapidus*, all living in moist habitats with rich grass cover, although *M. arvalis* is mainly associated to agricultural lands^54^. Specifically, *A. sapidus* is deemed a competitive species which can displace the Cabrera vole because of its larger size^35^. However, based on its current distribution and the fossil record, the two species seem to have segregated the spatial and temporal use of habitats^30,53,55^. Therefore, *A. sapidus* is not considered to be responsible for competitive displacement in France. Conversely, interspecific competition with the other microtines for a habitat specialist species like *M. cabrerae* could explain why Cabrera voles are not in the French territory. Consequently, biotic interactions may be having a large influence on the species’ current realized niche and thus, it could also be the case for the paleo-niches.

In conclusion, a combined environmental and geographical framework has enabled us to identify the lack of niche conservatism in the Cabrera vole. Therefore, forecastings of climate change should contemplate the non-niche conservatism approach and integrate other mechanisms of species, such as adaptation or preadaptation, rather than only global geographical migration towards higher latitudes. Based on our geographical approach, the species’ distribution should be limited to northern territories. However, the fossil record does not support these predictions. Our historical assessment clarifies the capabilities and limitations of this Iberian endemic vole from its own trajectory. Thus, *M. cabrerae* may have revealed some degree of adaptation to deal with climate changes throughout the wide time periods. Nevertheless, the adaptive ability of the species could be highly limited under arid conditions like MIS5e where the species reduced its environmental space notoriously. Therefore, a future climate change scenario developed in less than a century with warm temperatures like in MIS5e but drier might exceed the adaptive ability of Cabrera voles and range contraction could be the general rule. Notwithstanding, the realized niche shift could be also attributed to the non-equilibrium of species’ distribution with climate and the truncation of its fundamental niche. These alternatives should be analyzed in future studies using other approaches.

## Methods

### Environmental variables: current and past scenarios

The environmental framework was defined through a set of nineteen bioclimatic variables from WorldClim with 2.5 min (approx. 4.6 km × 4.6 km) spatial resolution^56^. To minimise multicolinearity in predictors, the variables were first arranged in a cluster dendrogram with Pearson correlations. One bioclimatic variable was selected for each branch (Pearson correlation: r <  ± 0.7) based on the biology of the species and the ability of the model to distinguish the regions of the environment and vole occurrences. The subset of variables was also filtered with a variance inflation factor: VIF < 5^57^ (R library HH^58^) to remove the predictors already explained by the rest of the variables. Finally, the explanatory variables were reduced according to the variable importance analyses from the conducted SDMs^25^. In the end, a set of five bioclimatic variables (Supplementary Fig. S4) was applied in all SDMs, PCAs as well as subsequent analyses.

The selected bioclimatic variables reflect the biological restrictions of the current distribution of *M. cabrerae*. The species is a specialist of grassland habitats with marked Mediterranean conditions^30^ and avoids the Euro-Siberian zone^59^, characteristics represented by the temperature seasonality variable (Bio 4). The other variables delimit suitable areas for the species in temperature in the wettest and driest quarters (Bio 8 and Bio 9), and precipitation in the warmest and coldest quarters (Bio 18 and Bio 19).

Temporal transferability was evaluated for 4 climate scenarios: current climatic conditions and 3 paleoclimatic scenarios corresponding to the Last Interglacial Period dated around 128-116 kyr. B.P. (MIS5e)^60^, the Last Glacial Maximum, 21 kyr. B.P. ago (inside the MIS2: 28-11.7 kyr. B.P.)^61–63^ and the Mid-Holocene, 6 kyr. B.P. ago (inside the MIS1: 11.7 kyr. B.P. to present time)^61,63,64^ (see the average values of bioclimatic variables in Supplementary Fig. S4). The LIP was a warm period at the beginning of the Late Pleistocene. The climate during MIS5e was highly continental with marked seasonality changes, and it was generally slightly dryer than the present with increasing precipitation from inland to marine areas^65^. In contrast, conditions towards the end of the Late Pleistocene were notoriously cold and arid due to the Last Glacial Maximum during MIS2. In spite of the harsh climate in Europe^62^ LGM in the Iberian Peninsula presented cold temperatures, but aridity levels were not very low. In fact, there were periods with relatively high water availability in the Mediterranean region^66^. The glacial landscapes were dominated by cold steppe formations with coniferous forest patches and mesophytes and thermophytes refuge areas^67^. The Mid-Holocene brought an improvement for the species in climate conditions, which were once again warmer and moister^66^. As a result, the Mediterranean region was covered by deciduous forests^68^.

Paleoclimate data were derived from the simulations of the Community Climate System Model (CCSM) global circulation model (GCM). CCSM was used for MIS5e data, while MIS1 and MIS2 were developed with CCSM4^56^.

### Occurrence and fossil record datasets

We obtained a total of 404 current occurrence records from the Spanish inventory of terrestrial species^69^, Mira, *et al*.^70^ and Garrido-García, *et al*.^51^ to establish the global distribution of *M. cabrerae*.

Fossil records were obtained from the species’ review by Laplana and Sevilla^28^ and completed with the records in Cuenca-Bescos, *et al*.^71^. Since fossil records were classified in wide periods, we only considered records with a chronology within the paleoclimate scenarios, spanning the whole period (Supplementary Table S1). A total of 28, 16 and 13 fossil records were used for MIS1, MIS2 and MIS5e, respectively.

To avoid statistical artefacts related to spatial autocorrelation, only one datum of the species was considered for each 10-km cell of the territorial grid, both in occurrence and fossil records.

### Niche modelling

Niche conservatism of *M. cabrerae* was evaluated using a combined methodological approach: a geographical analysis using SDMs and environmental assessment with several statistical tests of niche overlap. We compared geographical projections and environmental analyses of the current environment with the corresponding geographical projections and environmental analyses for each period in paleoclimate scenarios over the native territory. Thus, the analyses of the current distribution of Cabrera voles comprised environmental conditions of the Iberian Peninsula, while the modelling of the paleo-scenarios considered the environment of the Iberian Peninsula and the French region. All statistical analyses were carried out in R^72^.

#### Geographical analysis: Species Distribution Models

The geographical approach was based on the temporal transferability of SDMs. The SDMs were built with three robust modelling algorithms: Generalized linear model (GLM), RF (R library randomForest^73^) and Maximum Entropy (Maxent^74^) (R library dismo^75^). We specifically included GLM because of its flexibility to control all the factors involved in the model such as interactions or variable fitting, while Maxent and RF were used for their good predictive performance^7,76^. All models were built using current occurrence data of the species plus a background dataset (with 20% of the points in the global territory considered) for Maxent and weighted background for the GLM algorithm, whereas the RF and GLM were developed with a set of pseudoabsences. To achieve the best fit between the ecological response curves of the species and the modelled ones, we redefined the structure of the models, *e.g*. the GLM’s degree of the polynomials of the environmental variables or the features of Maxent. We also tried to minimize the complexity of the models. Thus, we conducted several Maxent models with different regularization parameters to finally fix this parameter at 3 units based on the criteria of the adjustment to ecological curves and gain values. Higher values of the regularization parameter prevent over-fitting model predictions to the presence locations values^77^. Model fit was assessed by explained variance for RF and GLM (called deviance or *D*^2^) and by gain for Maxent (closed to deviance of GLM), as both measure model quality^77,78^.

Finally, all models were evaluated by a bootstrapping validation procedure consisting of 20 times iterative data partitioning (40–60%, testing-training, respectively), using the AUC test. The AUC is a threshold-independent measurement that assesses the true positive fraction versus the false positive fraction of SDM predictions^79^, providing a single model performance score^74^.

Models were projected to the three paleoclimatic scenarios to predict Cabrera vole paleo-distribution, and thus identify a possible change in the climatic niche of the species. Niche predictions were assessed by the AUC test and the fossil record, an independent dataset of presence.

Similarly, all algorithms were calibrated for each of the paleoclimatic scenarios, following the previously described methodology, and transferred to the current climate scenario. The niche projections for the current climate framework were evaluated with the AUC test using the 404 points of current species occurrence.

The MoD of Maxent 3.3.3k software^74^ was conducted for projected variables from current to paleoclimatic scenarios. This test reveals the variables and regions where a projected variable falls outside the calibrated range of values.

#### Environmental analysis: PCA analysis

From an environmental perspective, we evaluated the degree of similarity or differentiation in the ecological niches throughout the species’ history.

First, the global climatic space from all scenarios (current and the three paleo-scenarios) were summarized in the two-dimensional space of the PCA (R library ade4^80^). In this framework, individual hyperspaces were created to represent the current and one of each paleo-niches of the species (repeated for the three paleo-scenarios). The environmental hyperspace of the occupied areas of the different scenarios was compared to analyze changes in the species niche. Niche overlap was assessed based on the Schoener’s D index, an ecological space-based metric which ranges between 0 (no niche overlap) and 1 (both niches have identical environmental spaces)^81^.

Following the methodology proposed by Warren, *et al*.^81^ and Broennimann, *et al*.^25^, we calculated two simulation-based tests to identify overlap patterns of current and past niches of Cabrera voles: the niche equivalency and the niche similarity tests (R library ecospat^82^). The niche equivalency test determines whether two niches are identical when their occurrences are reallocated. Species occurrences in the two temporal scenarios (the current and one of the paleoclimatic scenarios, successively) were pooled and randomly split 100 times into two datasets with the same number of data as in the original scenario to conduct new niche models and their respective D metrics. This random process allowed us to create a null distribution of D values for comparison with observed D. So, when the D value was inside the 95% confidence interval of the null distribution, we could assume that the niches were equivalent. The niche similarity test focuses on both the environment of the occurrences and the environment of the background dataset. Therefore, this test analyzes if niches are more similar or different from one another than expected by local similarities. In this case, one of the niches was created from randomly gathered occurrences in the available environment and compared to the other niche to determine the D metric of overlap. The process was iterated 100 times to obtain the null distribution of D values. So, when measured D was outside 95% of the simulated distribution, the species occupied areas more similar or different than expected by chance under both scenarios. As the niche similarity test was assessed in both directions (*i.e*. from the current climate to a paleoclimate scenario and vice versa).

Additionally, the niche dynamics of *M. cabrerae* in the different scenarios were evaluated with the indices of expansion, stability and unfilling (R library ecospat^82^). Niche expansion referred to conditions inside the paleo-niches that were not included in the current niche. On the contrary, unfilling represented conditions from the current distribution, which were not available in the paleo-niche. The stability index denoted common conditions throughout the niches of the Cabrera voles^24^.

### Data availability

Fossil record data are available in Supplementary Table S1 of Supplementary Information. All climate GIS layers are available as raster grids from the Worldclim database: www.worldclim.org/version1.

## Electronic supplementary material

Supplementary Information
