# Supplementary material for: Assessment of the effect of climate changes in the Late Pleistocene and Holocene on niche conservatism of an arvicolid specialist
Source: Sci Rep. 2018 Jun 28;8:9780. doi: 10.1038/s41598-018-28000-0 (PMC6023864; doi:10.1038/s41598-018-28000-0)
Supplement: Supplementary file 1 — Supplementary Information [file 41598_2018_28000_MOESM1_ESM.pdf]

Assessment of the effect of climate changes in the Late Pleistocene and Holocene on niche conservatism of an arvicolid specialist

Elena Castellanos-Frías, Nuria García, Emilio Virgós

SUPPLEMENTARY INFORMATION

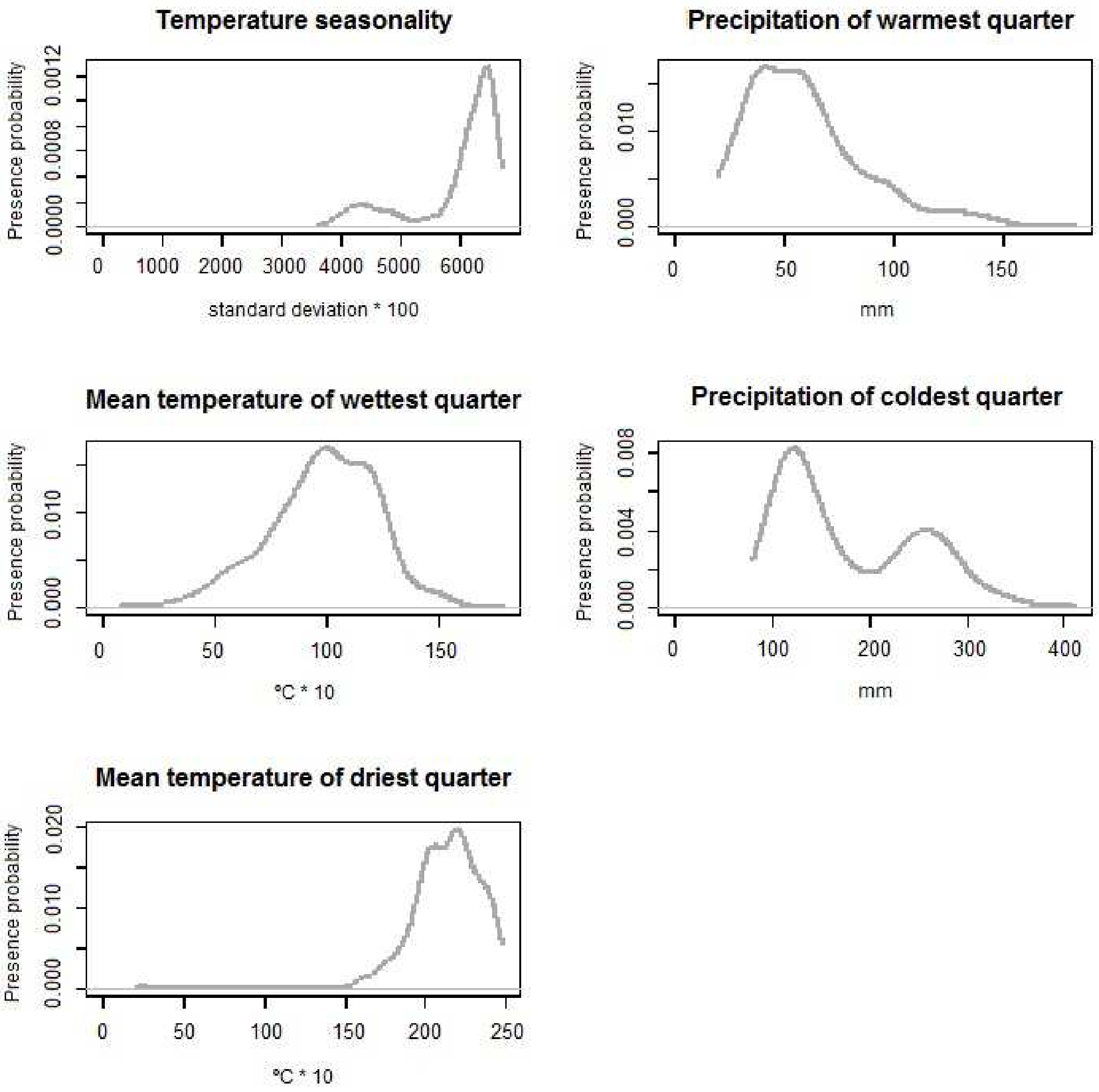

Supplementary Fig. S1. Response curves of *M. cabrerae* to bioclimatic variables in its current distribution.

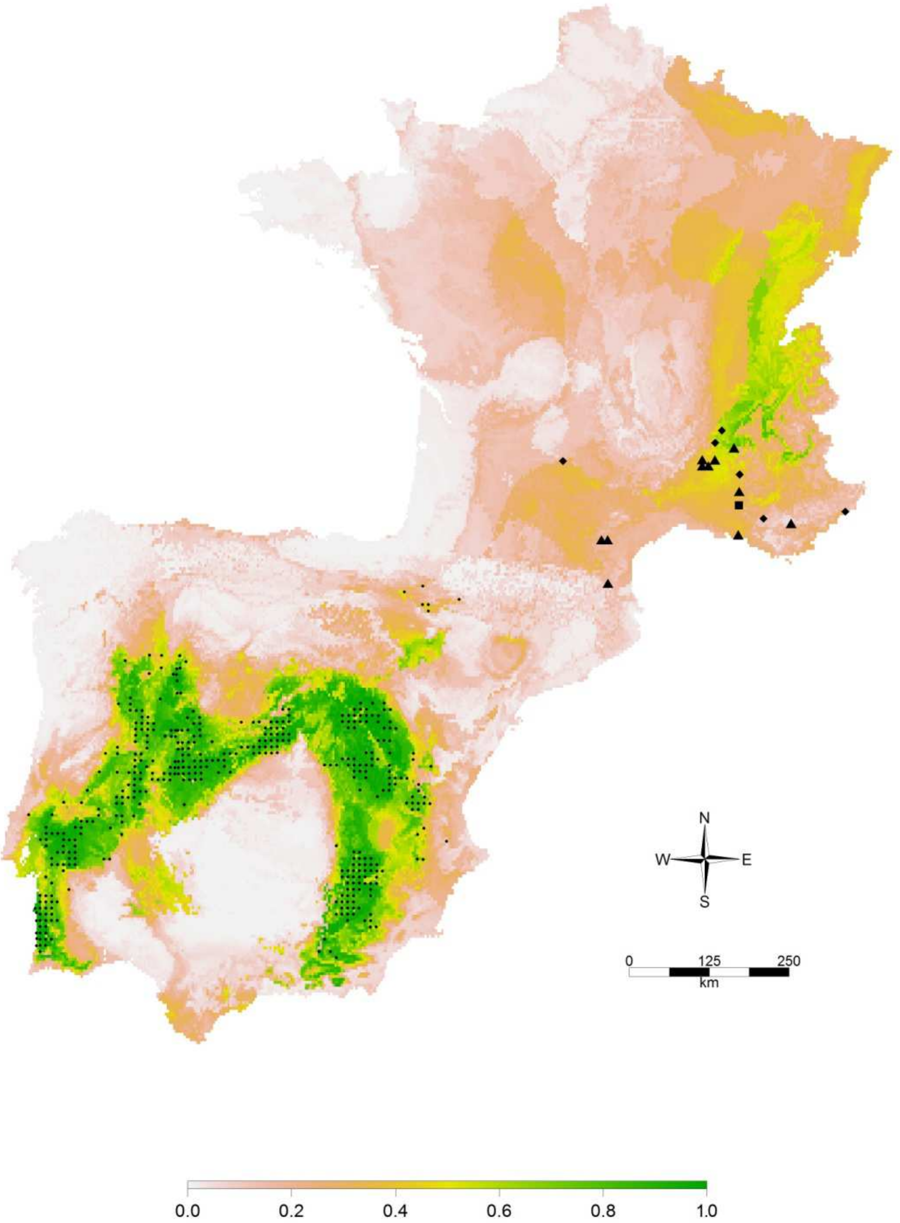

Supplementary Fig. S2. Suitability map of the spatial transferability of current SDM (Random forest model) for *M. cabrerae* beyond its distribution limits. Dots identify the (●) current occurrences, the fossil record of (▲) Holocene, (■) Last Glacial Maximum and (◆) Last Interglacial of the species. Figure was created in R v3.3.2[1] (https://www.R-project.org/).

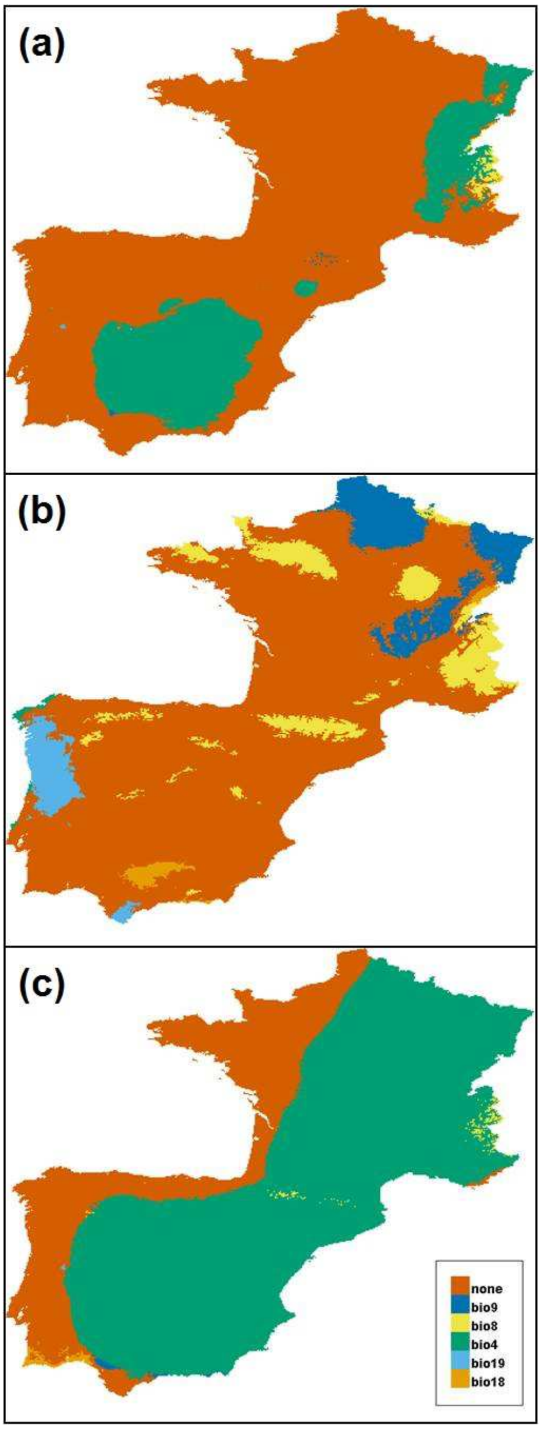

Supplementary Fig. S3. Most dissimilar variable analysis (MoD) of Maxent software across the three paleoclimatic scenarios: (a) MIS 1, (b) MIS 2 and (b) MIS 5e. The bioclimatic variables indicated in the maps represent areas where the values of the variable are outside the range used during model calibration. Model projection in these areas is executed keeping the values of climatic suitability as threshold values in the calibration. Figure was created in MaxEnt v3.3.3k[2] (http://rob.schapire.net/).

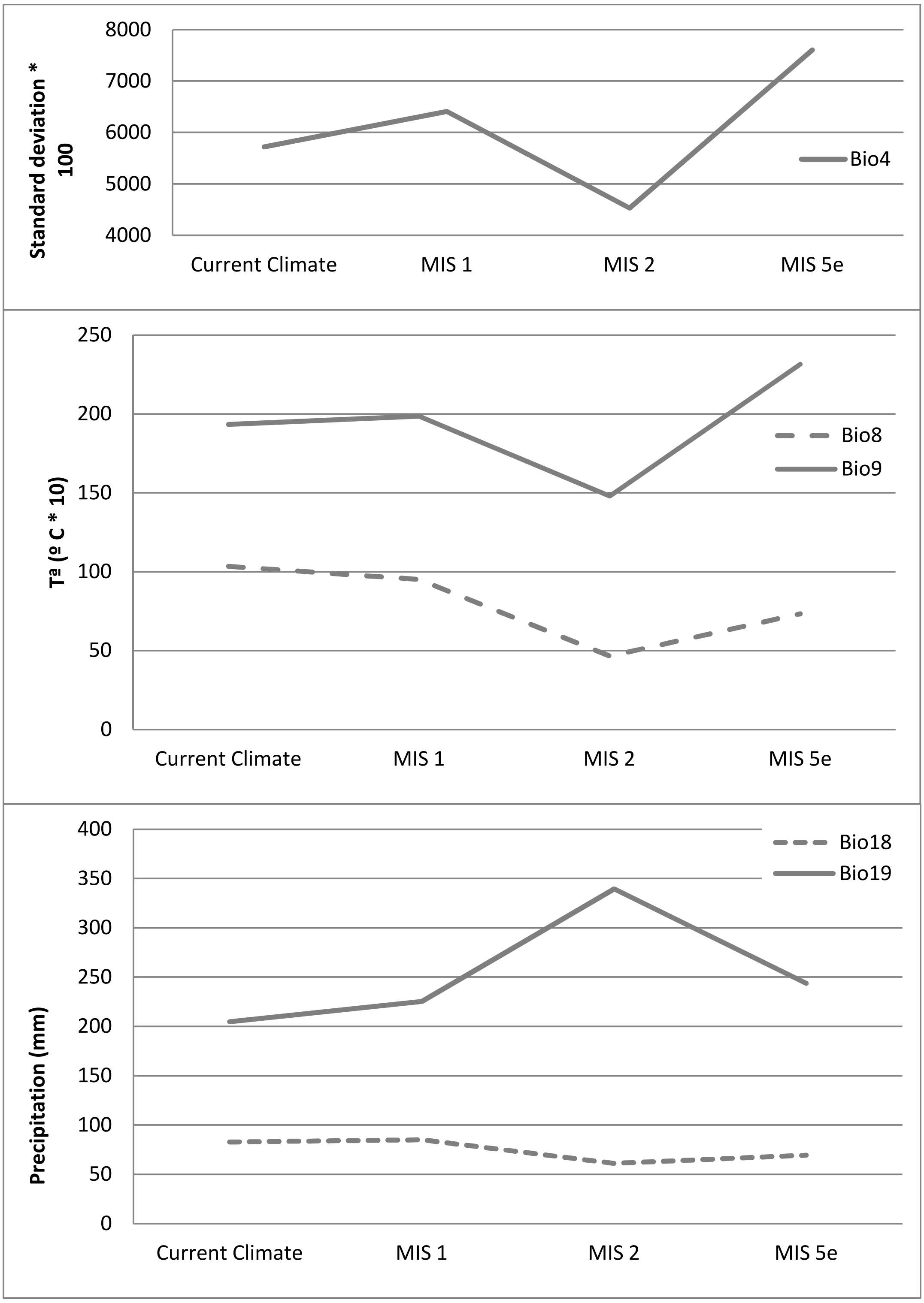

Supplementary Fig. S4. Average values of the bioclimatic variables: temperature seasonality (Bio 4), mean temperature of the wettest quarter (Bio 8), mean temperature of the driest quarter (Bio 9), precipitation in the warmest quarter (Bio 18) and precipitation in the coldest quarter (Bio 19) for the four climatic scenarios (Current climate, MIS1, MIS2 and MIS5e) in the common range of the Iberian Peninsula.

Supplementary Table S1. Fossil record of *Microtus cabrerae* derived from Laplana and Sevilla [3], and completed with Cuenca-Bescos, et al. [4].

Chronological filtering was performed on the original fossil record set to select the records within the considered climatic chronology.

Period	Country	Site	Chronology (kyr. B.P.)	Reference
MIS1	France	Combe Obscure	*ca*. 4.5 - 6.5	Brandy [5]; Helmer, et al. [6]
		Grotte de la Chauve-Souris	*ca*. 3.845 - 9.94	Jeannet and Vital [7]
		Baume d'Oullins	*ca*. 4.5 - 6.5	Helmer, et al. [6],Helmer and Vigne [8]
		Grotte de Fontcombe		Jeannet [9]
		Baume Claire and Baume Sourde	*ca*. 3.95 - 5.3	Jeannet [10]; Furestier [11]; Jeannet and Vital [7],Ducos [12]
		Abri de la Font-des-Pigeons		Ducos [12];Poitevin, et al. [13]
		Baume de Font-Brégoua		Brandy [5],Poitevin, et al. [13],Brandy [14]; Paunesco and Abbassi [15]; Paunesco and Brunet-Lecomte [16]
		Grotte d'Unang	*ca*. 4 - 6	Brandy [14]; Michaux [17]; Hervet [18]
		Grotte de l'Abeurador	*ca.* 6.26 ± 0.09	Marquet [19]; Marquet [20]; Vaquer and Ruas [21]
		Abri de Font-Juvénal	*ca*. 4.2 - 6.41 ± 0.1	Marquet [19]; Thiebault and Vernet [22]; Marquet [20]
		Caune Ouest	*ca*. 5.165 ± 0.125	Brochier, et al. [23]; MuséumNationald'HistoireNaturelle [24]
	Spain	Cova del Frare	*ca*. 4.45 - 6.38	Alcalde Gurt [25]; Albert, et al. [26]
		Cova del Pasteral	*ca*. 5.27 ± 0.07	Alcalde Gurt [25]; Gibaja, et al. [27]
		Cova 120	3 - 6	Alcalde Gurt [25]; Agustí, et al. [28]; Cucchi, et al. [29]; Cuenca-Bescos, et al. [4]
		Cova de l'Arbreda		Alcalde Gurt [25]; Alcalde Gurt [30]
		Cova Colomera	*ca*.6.87 - 7.06 ± 0.07	Bañuls-Cardona and López-García [31]; López-García and Cuenca-Bescós [32]; López-García, et al. [33]
		Cueva del Coscojar		Gil Bazán [34]; Fernández-Posse, et al. [35]
		Cueva de Chaves	*ca*. 6.12 - 6.77 ± 0.07	Utrilla, et al. [36]

		Cova de les Cendres	*ca*. 6 - 7.5	Guillem Calatayud [37]; Guillem Calatayud [38], Guillem Calatayud [39]; Guillem Calatayud [40]
		Cova de Bolumini		Guillem Calatayud [37], Guillem Calatayud [38]
		Abric de la Falguera	*ca*. 6.51 ± 0.07	Guillem Calatayud [41], Puchol and Tortosa [42]
		Cova de la Sarsa-Sector II: Gatera	*ca*. 6.9	Sevilla [43], López-García and Cuenca-Bescós [44]
		Abric del Mas Martí	*ca*. 6.5	Fernández López de Pablo, et al. [45]
		Cingle del Mas Cremat	*ca*. 6.74 - 6.99	Vicente Gabarda, et al. [46], Guillem Calatayud [47]
		Cova Fosca	*ca*. 6.2	Sesé [48]
		Cueva de la Ventana	*ca*. 6.01 - 6.56 ± 0.04	Sánchez, et al. [49], Doce, et al. [50]
		Cueva de El Toro	*ca*. 4.22 - 4.33	Mederos Martín [51], Watson, et al. [52]
		Cueva del Nacimiento	*ca*. 4 - 6	López Martínez and Sanchiz [53]
**MIS2**	France	Abri de Soubeyras	*ca*. 15.075 - 18.27	Crégut-Bonnoure, et al. [54], Brochier [55]
	Spain	Cova de l'Arbreda	*ca*. 19.48	Alcalde Gurt [25], Alcalde Gurt [30], Alcalde Gurt, et al. [56]
		Cueva de Valdavara I	*ca*. 17.89	López-García, et al. [57], Vaquero Rodríguez, et al. [58], Bañuls-Cardona, et al. [59]
		El Portalón	*ca*. 16.89 - 30.3	López-García, et al. [60], López-García [61]
		Cova de les Cendres	*ca*. 21.23	Guillem Calatayud [37], Guillem Calatayud [39], Villaverde and Roman [62], Villaverde, et al. [63], Villaverde, et al. [64]
		Cova Negra	*ca*. 21	Guillem Calatayud [37], Guillem Calatayud [39]
		Sala de las Chimeneas	*ca*. 21.63 - 21.27	Bañuls-Cardona, et al. [59], Bañuls and López-García [65], Bañuls, et al. [66]
		Cueva de la Carihuela	*ca*. 18.8 - 22.4	Ruiz Bustos [67], Fernández, et al. [68]
		Higueral de Valleja Cave	*ca*. 20.78	Turner, et al. [69], Jennings, et al. [70]
		Cueva de la Pastora I		Ruiz Bustos [71], Ruiz Bustos [72]
		Cueva de los Ojos	*ca*. 18 - 22	Fuentes Jiménez [73]
		Cueva de Nerja	*ca*. 17.5 - 24	Cortés-Sánchez, et al. [74]
		Cova del Toll	*ca*. 13 - 35	Fernández-García and López-García [75], Fernández-García [76]

		Cova Fosca	*ca*. 19	Cuenca-Bescos, et al. [4], Alcalde Gurt [25]
	Portugal	Grotte de Caldeirao	*ca*. 18 - 30	Póvoas [77], Povoas, et al. [78]
	United Kingdom	Gorham's Cave	*ca*. 18.4	Denys [79], Cuenca-Bescós, et al. [80], López-García, et al. [81], Price [82]
**MIS5e**	France	Grotte de Payre	*ca*. 105 - 135	Desclaux, et al. [83], Rivals, et al. [84]
		Baume Moula Guercy	*ca*. 100 - 120	Desclaux and Defleur [85], Defleur, et al. [86], Defleur, et al. [87]
		Grand Abri aux Puces	*ca*. 117 - 127	Slimak, et al. [88]
		Grotte de l'Adaouste		Defleur, et al. [89]
		Grotte de Le Lazaret	*ca*. 130	Valensi, et al. [90], Hanquet, et al. [91]
		Coudoulous II	*ca*. 112 ± 35 - 140	Couchoud [92], Cochard [93]
	Spain	Cova de Bolomor	*ca*. 121 ± 18	Guillem Calatayud [37], Guillem Calatayud [39], Fernández Peris, et al. [94]
		Cova Negra	*ca*. 117 ± 17	Guillem Calatayud [37], Guillem Calatayud [39], Fernández Peris, et al. [94], Perez Ripoll [95], Arsuaga, et al. [96]
		Cueva de las Pinturas		Sesé and Ruiz Bustos [97]
		Cueva del Camino	*ca*. 74 - 140	Toni and Molero [98], Arsuaga, et al. [99], Arsuaga, et al. [100], Arsuaga, et al. [101], Blain, et al. [102]
		Preresa	*ca*. 107 - 122.1 ± 11.1	Sesé, et al. [103]
		Cueva de la Carihuela		Ruiz Bustos [67], Vega Toscano [104]
	Portugal	Goldra	*ca*. 125	Antunes, et al. [105]

REFERENCES

1 R Core Team. *R: A language and environment for statistical computing*, <http://www.R-project.org> (2015).

2 Phillips, S. J., Anderson, R. P. & Schapire, R. E. Maximum entropy modeling of species geographic distributions. *Ecol. Model.* **190**, 231-259, doi:10.1016/j.ecolmodel.2005.03.026 (2006).

3 Laplana, C. & Sevilla, P. Documenting the biogeographic history of *Microtus cabrerae* through its fossil record. *Mammal Rev.* **43**, 309-322 (2013).

4 Cuenca-Bescos, G. *et al.* Pleistocene history of Iberomys, an endangered endemic rodent from southwestern Europe. *Integrative Zoology* **9**, 481-497 (2014).

5 Brandy, L. Les rongeurs de quelques grottes du Würm récent et du Postglaciaire en Provence et en Languedoc. *Supplément du Bulletin de l'Association Française pour l'Étude du Quaternaire* **47**, 347-351 (1977).

6 Helmer, D., Gourichon, L., Sidi Maamar, H. & Vigne, J.-D. L'élevage des caprinés néolithiques dans le sud-est de la France: saisonnalité des abattages, relations entre grottes-bergeries et sites de plein air. *Anthropozoologica* **40**, 167-189 (2005).

7 Jeannet, M. & Vital, J. La grotte de la Chauve-Souris à Donzère (Drôme, France): L'environnement holocène par la méthode quantitative appliquée aux microvertébrés. Essai de définition et application. *Revue de Paléobiologie* **28**, 139-173 (2009).

8 Helmer, D. & Vigne, J.-D. Was milk a "secondary product" in the Old World Neolithisation process? Its role in the domestication of cattle, sheep and goats. *Anthropozoologica* **42**, 9-40 (2007).

9 Jeannet, M. Les rongeurs des niveaux néolithiques de la grotte de Saint-Marcel (Ardèche). *Ardèche Archéologie* **5**, 46 (1988).

10 Jeannet, M. Biometrie et ecologie de Microtus brecciensis (Mammalia, Rodentia). *Revue de Paléobiologie* **19**, 339-357 (2000).

11 Furestier, R. *Les industries lithiques campaniformes du sud-est de la France*, Université de Provence-Aix-Marseille I, (2005).

12 Ducos, P. Le gisement de Chateauneuf-les-Martigues (Bouches-du-Rhône). Les mammifères et les problèmes de la domestication. *Bulletin du Musée d'Anthropologie préhistorique de Monaco* **5** (1958).

13 Poitevin, F., Bayle, P. & Courtin, J. Mise en place des faunes de micromammifères (rongeurs, insectivores) dans la région méditerranéenne française au Post-glaciaire. *Vie Milieu* **40**, 144-149 (1990).

14 Brandy, L. D. Etude d'une population de Microtus brecciensis en Provence; relations avec les Campagnols méditerranéens actuels. *Rapport sur le stage de paléontologie effectué au Laboratoire d'Evolution des Vertébrés* (1976).

15 Paunesco, A. C. & Abbassi, M. Les rongeurs (Rodentia, Mammalia) de la grotte de Fontbrégoua (Var, France). *Travaux de l'Institut de Spéologie Emile Racovitza* **37-38**, 247-300 (1999).

16 Paunesco, A.-C. & Brunet-Lecomte, P. Analyse odontométrique du sous-genre Microtus (Iberomys) Chaline, 1972 (Rodentia, Arvicolinae) du sud de la France au Pléistocène moyen et supérieur. *Bulletin du Musée d'anthropologie préhistorique de Monaco*, 31-39 (2005).

17 Michaux, J. in *La grotte d'Unang à Malemort-du-Comtat* (eds Maurice Paccard & Claude Bouville) (Service d'archéologie de Vaucluse, 1993).

18 Hervet, S. Tortues du Quaternaire de France: critères de détermination, répartitions chronologique et géographique. *Mésogée* **58**, 3-47 (2000).

19 Marquet, J. Les modifications de l'environnement Postglaciaire en France méditerranéenne d'après les rongeurs de l'Abeurador et de Font-Juvenal. *Premières communautés paysannes en méditerranée occidentale. Paris: Comite National de la Recherche Scientifique*, 155-163 (1987).

20 Marquet, J. C. Paléoenvironnement et chronologie des sites du domaine atlantique français d'âge Pléistocène Moyen et Supérieur d'après l'étude des rongeurs. *Les cahiers de la claise* **2**, 1-345 (1993).

21 Vaquer, J. & Ruas, M.-P. La grotte de l'Abeurador Félines-Minervois (Hérault): occupations humaines et environnement du Tardiglaciaire à l'Holocène. *De Méditerranée et d'Ailleurs. Mélanges Offerts à Jean Guilaine*, 761-792 (2009).

22 Thiebault, S. & Vernet, J.-L. Végétations méditerranéennes et civilisations préhistoriques: le cas de Font-Juvénal. *Bulletin de la Société Botanique de France. Actualités Botaniques* **139**, 441-450 (1992).

23 Brochier, J. E., Claustre, F. & Heinz, C. Environmental impact of Neolithic and Bronze Age farming in the eastern Pyrenees forelands, based on multidisciplinary investigations at La Caune de Bélesta (Bélesta Cave), near Perpignan, France. *Vegetation History and Archaeobotany* **7**, 1-9 (1998).

24 MuséumNationald'HistoireNaturelle. *National inventory of natural heritage*, <http://inpn.mnhn.fr > (2003-2012).

25 Alcalde Gurt, G. *Les faunes de Rongeurs du Pléistocène Supérieur et de l'Holocène de Catalogne (Espagne) et leurs significations paléoécologiques et paléoclimatiques*, (1986).

26 Albert, R. M., Mangado, X. & Martin, A. in *First European Meeting on Phytolith Research.* 187 (Editorial CSIC-CSIC Press).

27 Gibaja, J. F., Majó, T., Chambon, P., Ruiz, J. & Subirà, M. E. Neolithic Funeral rituals: child burials in the Northeastern Iberian Peninsula. *Complutum* **21**, 47-68 (2010).

28 Agustí, B. *et al.* El yacimiento del paleolítico medio de la Cueva 120 (La Garrotxa, Cataluña). Primeros resultados. *Cuaternario y Geomorfología* **1**, 1-13 (1987).

29 Cucchi, T., Vigne, J. D. & Auffray, J. C. First occurrence of the house mouse (Mus musculus domesticus Schwarz & Schwarz, 1943) in the Western Mediterranean: a zooarchaeological revision of subfossil occurrences. *Biol. J. Linnean Soc.* **84**, 429-445 (2005).

30 Alcalde Gurt, G. Els rosegadors del Paleolític Superior de la cova de l'Arbreda (Serinyà, Catalunya): significació paleoecològica i paleoclimàtica. *Cypsela* **6**, 89-96 (1987).

31 Bañuls-Cardona, S. & López-García, J. Análisis de los cambios paleoambientales del Pleistoceno superior final-Holoceno a partir del estudio de micromamíferos de la Cova Colomera (Sant Esteve de la Sarga, Lleida). *II Jornadas de Jóvenes en Investigación Arqueológica,(Madrid, 6, 7 y 8 de mayo de 2009)* **2**, 475-478 (2009).

32 López-García, J. M. & Cuenca-Bescós, G. Evolution climatique durant le Pléistocène Supérieur en Catalogne (Nord-est de l'Espagne) d'après l'étude des micromammifères. *Quaternaire. Revue de l'Association française pour l'étude du Quaternaire* **21**, 249-257 (2010).

33 López-García, J. M. *et al.* First fossil evidence of an "interglacial refugium" in the Pyrenean region. *Naturwissenschaften* **97**, 753-761 (2010).

34 Gil Bazán, E. Estudio de los micromamíferos (Rodentia) de la Cueva del Coscojar, Mora de Rubielos, Teruel. *Teruel: Revista del Instituto de Estudios Turolenses*, 37-57 (1985).

35 Fernández-Posse, M. D., Gilman, A. & Martín, C. Consideraciones cronológicas sobre la Edad del Bronce en La Mancha. *Homenaje al profesor Manuel Fernández-Miranda* **6**, 111 (1996).

36 Utrilla, P., Lorenzo, J., Baldellou, V., Sopena, M. C. & Ayuso, P. in *IV Congreso del Neolítico Peninsular (Alicante 2006).* 131-140.

37 Guillem Calatayud, P. Paleontología continental: microfauna. *El Cuaternario del País Valenciano* **227**, 233 (1995).

38 Guillem Calatayud, P. Los micromamíferos (Rodentia, Insectivora y Chiroptera) de la secuencia Holocena de la Cova de les Cendres y Cova Bolumini. *Saguntum-PLAV,(Extra-2)*, 31-36 (1999).

39 Guillem Calatayud, P. in *De neandertales a cromañones: el inicio del poblamiento humano en las tierras valencianas* (ed Valentín Villaverde) 57-72 (Univ. de València, 2001).

40 Guillem Calatayud, P. in *La Cova de Les Cendres.* 189-194 (Museo Arqueológico de Alicante-MARQ).

41 Guillem Calatayud, P. in *El abric de la Falguera (Alcoi, Alacant): 8.000 años de ocupación humana en la cabecera del río de Alcoi.* 158-167 (Museo Arqueológico de Alicante-MARQ).

42 Puchol, O. G. & Tortosa, J. E. A. *El abric de la Falguera (Alcoi, Alacant): 8.000 años de ocupación humana en la cabecera del río de Alcoi*. (Museo Arqueológico de Alicante-MARQ, 2006).

43 Sevilla, P. Estudio paleontológico de los Quirópteros del Cuaternario español. *Paleontologia i evolució* **22**, 113-233 (1988).

44 López-García, J. M. & Cuenca-Bescós, G. Changes in the geographical distribution of *Microtus* (*Iberomys*) *cabrerae* (Thomas, 1906) from the Late Pleistocene to the Holocene. *Spanish Journal of Palaeontology* **27**, 117-124 (2012).

45 Fernández López de Pablo, J., Guillem Calatayud, P., Martínez-Valle, R. & Pérez-Milián, R. in *Actas del III Congreso del Neolítico en la Península Ibérica.* (eds R. Ontañón Peredo, C. García-Moncó Piñeiro, & P. Arias Cabal) 879-890.

46 Vicente Gabarda, M., Martínez Valle, R., Guillem Calatayud, P. & Iborra Eres, M. P. El Cingle del Mas Cremat (Portell de Morella, Castelló). Un asentamiento en altura, con ocupaciones del Mesolítico reciente. *Monografías Arqueológicas (Prehistoria)* **44**, 361-374 (2009).

47 Guillem Calatayud, P. in *El Cingle del Mas Cremat (Portell de Morella, Castellón): un asentamiento en altura con ocupaciones del Mesolítico Reciente al Neolítico Final* (ed D. Vizcaíno) 129-144 (Parque Eólico de las Cabrillas/Zona III del Plan Eólico Valenciano, 2010).

48 Sesé, C. Micromamíferos (Erinaceomorfos y Roedores) del final del Pleistoceno Superior y primera parte del Holoceno de Cova Fosca (Alto Maestrazgo, Castellón): Reconstrucción paleoambiental del entorno del yacimiento. *Archaeofauna*, 119-156 (2011).

49 Sánchez, A. *et al.* in *III Congreso del Neolítico en la Península Ibérica. Monografias del Instituto Internacional de Investigaciones Prehistóricas de Cantabria.* 155-165.

50 Doce, E. G., Sánchez, P. J. C., García, J. F. F., Magdaleno, P. Z. & Plaza, S. L. El marco cronológico de la neolitización en el sector sudoccidental de la submeseta norte española: Dataciones absolutas del yacimiento de la Atalaya (Muñopepe, Ávila). *Rubricatum: revista del Museu de Gavà*, 517-524 (2012).

51 Mederos Martín, A. La cronología absoluta de Andalucía Occidental durante la Prehistoria Reciente (6100-850 AC). *Spal. Revista de Prehistoria y Arqueología (1996, Vol. 5, p. 45-86)* (1996).

52 Watson, J., Paz, M., Tusell, M. & Mañosa, M. Análisis arqueofaunístico. Campaña de 1988. *La Cueva de El Toro (Sierra de El Torcal-Antequera-Málaga). Un Modelo de Ocupación Ganadera en el Territorio Andaluz Entre el VI y II Milenios ANE, Arqueología Monografías* **21**, 215-266 (2004).

53 López Martínez, N. & Sanchiz, F. Notas sobre los microvertebrados del yacimiento arqueológico de Pontones (Jaén). *Trabajos de Prehistoria* **38**, 134-138 (1981).

54 Crégut-Bonnoure, E. *et al.* The karst of the Vaucluse, an exceptional record for the Last Glacial Maximum (LGM) and the Late-glacial period palaeoenvironment of southeastern France. *Quaternary International* **339**, 41-61 (2014).

55 Brochier, J. É. *Evolution des climats et des paysages vauclusiens au cours du Würmien récent et du Postglaciaire*. Vol. 1 (Éditions du Centre National de la Recherche Scientifique, Centre Régional de Publication, 1977).

56 Alcalde Gurt, G., Vila i Mitjà, A. & Estévez Escalera, J. Algunes precisions sobre l'estratigrafia de la Cova de l'Arbreda (Serinyà, Girona). *Revista de Girona* **96**, 189-193 (1981).

57 López-García, J. M. *et al.* Small vertebrates (Amphibia, Squamata, Mammalia) from the late Pleistocene-Holocene of the Valdavara-1 cave (Galicia, northwestern Spain). *Geobios* **44**, 253-269 (2011).

58 Vaquero Rodríguez, M. *et al.* New radiometric dates for the Prehistory of Northwestern Iberia: Valdavara Cave (Becerreá, Lugo). *Trabajos de Prehistoria* **66**, 99-113 (2009).

59 Bañuls-Cardona, S., López-García, J. M., Blain, H.-A., Lozano-Fernández, I. & Cuenca-Bescós, G. The end of the Last Glacial Maximum in the Iberian Peninsula characterized by the small-mammal assemblages. *Journal of Iberian Geology* **40**, 19-27 (2014).

60 López-García, J. M. *et al.* Palaeoenvironmental and palaeoclimatic reconstruction of the latest Pleistocene of El Portalón site, Sierra de Atapuerca, northwestern Spain. *Palaeogeography, Palaeoclimatology, Palaeoecology* **292**, 453-464 (2010).

61 López-García, J. M. 416 (Editorial Académica Española, 2011).

62 Villaverde, V. & Roman, D. Avance al estudio de los niveles gravetienses de la Cova de les Cendres. Resultados de la excavación del sondeo (cuadros A/B/C-17) y su valoración en el contexto del Gravetiense mediterráneo ibérico. *Archivo de Prehistoria Levantina* **25**, 19-59 (2004).

63 Villaverde, V. *et al.* Els nivells magdalenians de la Cova de les Cendres (Teulada, Moraira). Resultats del sondeig del quadre A-17. (1997).

64 Villaverde, V. *et al.* El Paleolítico superior en el País Valenciano. Novedades y perspectivas. *Jornadas Internacionales sobre el Paleolítico superior peninsular. Novedades del Siglo XXI. Monografies del SERP* **8**, 85-113 (2010).

65 Bañuls, S. & López-García, J. M. Nota preliminar sobre el estudio de los roedores del Pleistoceno superior de la Sala de las Chimeneas de la Cueva de Maltravieso (Cáceres, Extremadura). *Cidaris*, 67-72 (2010).

66 Bañuls, S., López-García, J. M., Blain, H.-A. & Salomó, A. C. Climate and landscape during the Last Glacial Maximum in southwestern Iberia: The small-vertebrate association from the Sala de las Chimeneas, Maltravieso, Extremadura. *Comptes Rendus Palevol* **11**, 31-40 (2012).

67 Ruiz Bustos, A. Estudio paleoecológico de los sedimentos con presencia del hombre de Neandertal en la Cueva de la Carihuela (Piñar, Granada). *Ayuntamiento de Píñar (Granada, Spain)* (2000).

68 Fernández, S. *et al.* The Holocene and Upper Pleistocene pollen sequence of Carihuela cave, southern Spain. *Geobios* **40**, 75-90 (2007).

69 Turner, E. *et al.* Larger vertebrate remains from the Middle to Upper Palaeolithic occupations of Higueral de Valleja Cave, southern Spain. *Hugo Obermaier-Gesellschaft für Erforschung des Eiszeitalters und der Steinzeit e* **V**, 32-35 (2008).

70 Jennings, R. *et al.* New dates and palaeoenvironmental evidence for the Middle to Upper Palaeolithic occupation of Higueral de Valleja Cave, southern Spain. *Quat. Sci. Rev.* **28**, 830-839 (2009).

71 Ruiz Bustos, A. Parameterization of the distribution of arvicolid tooth enamel. *Acta Zoologica Cracoviensia* **39**, 435-446 (1996).

72 Ruiz Bustos, A. Aportaciones de las faunas de mamíferos a la bioestratigrafía y paleoecología de la cuenca de Guadix y Baza. *Estructura, tectónica activa, sismicidad, geomorfología y dataciones existentes*, 11-27 (2007).

73 Fuentes Jiménez, J. M. in *Resúmenes de las V Jornadas de Paleontología.*

74 Cortés-Sánchez, M. *et al.* Palaeoenvironmental and cultural dynamics of the coast of Málaga (Andalusia, Spain) during the Upper Pleistocene and early Holocene. *Quat. Sci. Rev.* **27**, 2176-2193 (2008).

75 Fernández-García, M. & López-García, J. Resultados preliminares del estudio de los roedores del Pleistoceno Superior-Holoceno de la cueva del Toll (Moià, Barcelona). *Paleodiversity and Palaeoecology of Iberian Ecosystems* (2012).

76 Fernández-García, M. Paleoecología y biocronología mediante el estudio de los roedores del Pleistoceno Superior-Holoceno de la cueva del Toll (Moià, Cataluña, NE de la península Ibérica). *Treballs del Museu de Geologia de Barcelona* **20**, 73-97 (2014).

77 Póvoas, L. Faunes de rongeurs actuelles et du Pléistocène supérieur au Portugal: les évidences des sites de Avecasta et Caldeirão. *Memórias e Notícias*, 275-283 (1991).

78 Povoas, L., Zilhao, J., Chaline, J. & Brunet-Lecomte, P. La faune de rongeurs du Pléistocene supérieur de la grotte de Caldeirao (Tomar, Portugal). *Quaternaire* **3**, 40-47 (1992).

79 Denys, C. Small mammals of Ibex Cave: biostratigraphical and palaeoecological remarks. *Gibraltar during the Quaternary: the southernmost part of Europe in the last two million years. Gibraltar Government Heritage Publications Monographs* **1**, 215-225 (2000).

80 Cuenca-Bescós, G., López-García, J. M. & Finlayson, C. in *XXIII Jornadas de Paleontología-Libro de resúmenes.* (eds J.C. Braga, A. Checa, & M. Company).

81 López-García, J. M., Cuenca-Bescós, G., Finlayson, C., Brown, K. & Pacheco, F. G. Palaeoenvironmental and palaeoclimatic proxies of the Gorham's cave small mammal sequence, Gibraltar, southern Iberia. *Quaternary International* **243**, 137-142 (2011).

82 Price, C. in *Neanderthals in Context: a report of the 1995–1998 excavations at Gorham's and Vanguard Caves, Gibraltar* 128-140 (2012).

83 Desclaux, E., El Hazzazi, N., Villette, P. & Dubar, M. Le Contexte environnemental des occupations humaines: l'apport de la microfaune, des restes aviaires et de la malacofaune. *Mémoire de la Societe Prehistorique Francaise* **46**, 91-105 (2008).

84 Rivals, F., Schulz, E. & Kaiser, T. M. Late and middle Pleistocene ungulates dietary diversity in Western Europe indicate variations of Neanderthal paleoenvironments through time and space. *Quat. Sci. Rev.* **28**, 3388-3400 (2009).

85 Desclaux, E. & Defleur, A. Étude préliminaire des micromammifères de la Baume Moula-Guercy à Soyons (Ardèche, France). Systématique, biostratigraphie et paléoécologie. *Quaternaire* **8**, 213-223 (1997).

86 Defleur, A., Crégut-Bonnoure, É., Desclaux, E. & Thinon, M. Présentation paléo-environnementale du remplissage de la Baume Moula-Guercy à Soyons (Ardèche): implications paléoclimatiques et chronologiques. *L'Anthropologie* **105**, 369-408 (2001).

87 Defleur, A., White, T., Valensi, P., Slimak, L. & Cregut-Bonnoure, E. Neanderthal cannibalism at Moula-Guercy, Ardeche, France. *Science* **286**, 128-131 (1999).

88 Slimak, L. *et al.* Le Grand Abri aux Puces, a Mousterian site from the Last Interglacial: paleogeography, paleoenvironment, and new excavation results. *Journal of Archaeological Science* **37**, 2747-2761 (2010).

89 Defleur, A. *et al.* Le niveau moustérien de la grotte de l'Adaouste (Jouques, Bouches-du-Rhône). Approche culturelle et paléoenvironnements. *Bulletin du Musée d'Anthropologie préhistorique de Monaco* **37**, 11-48 (1994).

90 Valensi, P. *et al.* Cadre climatique et environnemental des acheuléens de la grotte du Lazaret, à Nice. Données paléontologiques, biogéochimiques et radiométriques établies sur les faunes de vertébrés et d'invertébrés. *ArcheoSciences* **31**, 137-150 (2007).

91 Hanquet, C. *et al.* Caractérisation du climat et de la biodiversité au Pléistocène moyen final, d'après les faunes de vertébrés de la grotte du Lazaret (Nice, France). *Quaternaire. Revue de l'Association française pour l'étude du Quaternaire* **21**, 215-226 (2010).

92 Couchoud, I. *Etude pétrographique et isotopique des spéléothèmes du sud-ouest de la France formés en contexte archéologique: Contribution à la connaissance des paléoclimats régionaux du stade isotopique 5*, Université Sciences et Technologies-Bordeaux I, (2006).

93 Cochard, D. *Les léporidés dans la subsistance paléolithique du Sud de la France*, Université Sciences et Technologies-Bordeaux I, (2004).

94 Fernández Peris, J., Barciela, V., Blasco, R., Cuartero Monteagudo, F. & Sañudo, P. El Paleolítico Medio en el territorio valenciano y la variabilidad tecno-económica de la Cova del Bolomor. *Treballs d'Arqueologia*, 141-169 (2008).

95 Perez Ripoll, M. Los mamiferos del yacimiento musteriense de Cova Negra (Jativa, Valencia).(Les mammifères du site moustérien de CN (JV). *Servicio de Investigacion Prehistorica. Serie de Trabajos Varios Valencia* **53**, 7-147 (1977).

96 Arsuaga, J. L. *et al.* New Neandertal remains from Cova Negra (Valencia, Spain). *Journal of Human evolution* **52**, 31-58 (2007).

97 Sesé, C. & Ruiz Bustos, A. Nuevas faunas de micromamíferos del Pleistoceno del Norte de la Provincia de Madrid (España). *Boletin de la Real Sociedad Española de Historia Natural (Geología)* **87**, 115-139 (1992).

98 Toni, I. & Molero, G. in *Actas de las IV Jornadas de Paleontología* 359-373 (Salamanca, 1990).

99 Arsuaga, J. L. *et al.* El yacimiento arqueopaleontológico del Pleistoceno Superior de la Cueva del Camino en el Calvero de la Higuera (Pinilla del Valle, Madrid). *Zona arqueológica* **13**, 421-442 (2010).

100 Arsuaga, J. L., Baquedano, E. & Pérez-González, A. in *Proceedings of the XV World Congress of the International Union for Prehistoric and Protohistoric Sciences.* (ed L. Oosterbeek) 111-119 (BAR International Series).

101 Arsuaga, J. L. *et al.* Understanding the ancient habitats of the last-interglacial (late MIS 5) Neanderthals of central Iberia: Paleoenvironmental and taphonomic evidence from the Cueva del Camino (Spain) site. *Quaternary International* **275**, 55-75 (2012).

102 Blain, H.-A. *et al.* MIS 5/4 transition in a mountain environment: herpetofaunal assemblages from Cueva del Camino, central Spain. *Boreas* **43**, 107-120 (2014).

103 Sesé, C., Rubio-Jara, S., Panera, J. & Pérez-González, A. Micromamíferos del Pleistoceno Superior del yacimiento de PRERESA en el valle del Manzanares y su contribución a la reconstrucción paleoambiental de la cuenca de Madrid durante el Pleistoceno. *Estudios geológicos* **67**, 471-494 (2011).

104 Vega Toscano, L. G. in *Euromam. Excursion to the Guadix-Baza basin.* 43-53.

105 Antunes, M. T., Manupella, G., Mein, P. & Zbyszewski, G. Goldra: premier gisement karstique en Algarve, fauna et industries. *Ciências da Terra* **8**, 31-42 (1986).
